# Outcome from Complicated versus Uncomplicated Mild Traumatic Brain Injury

**DOI:** 10.1155/2012/415740

**Published:** 2012-04-19

**Authors:** Grant L. Iverson, Rael T. Lange, Minna Wäljas, Suvi Liimatainen, Prasun Dastidar, Kaisa M. Hartikainen, Seppo Soimakallio, Juha Öhman

**Affiliations:** ^1^Department of Psychiatry, University of British Columbia, 2255 Wesbrook Mall, Vancouver, BC, Canada V6T 2A1; ^2^Department of Orthopaedics and Rehabilitation, Walter Reed National Military Medical Center and Defense and Veterans Brain Injury Center, 11300 Rockville Pike, Suite 1100, North Bethesda, MD 20852, USA; ^3^Department of Neurosciences and Rehabilitation, Tampere University Hospital and University of Tampere Medical School, PL 2000, 33521 Tampere, Finland; ^4^Department of Neurosciences and Rehabilitation and Emergency Department Acuta, Tampere University Hospital, PL 2000, 33521 Tampere, Finland; ^5^Medical Imaging Centre of Pirkanmaa Hospital District and University of Tampere Medical School, PL 2000, 33521 Tampere, Finland

## Abstract

*Objective*. To compare acute outcome following complicated versus uncomplicated mild traumatic brain injury (MTBI) using neurocognitive and self-report measures. *Method*. Participants were 47 patients who presented to the emergency department of Tampere University Hospital, Finland. All completed MRI scanning, self-report measures, and neurocognitive testing at 3-4 weeks after injury. Participants were classified into the complicated MTBI or uncomplicated MTBI group based on the presence/absence of intracranial abnormality on day-of-injury CT scan or 3-4 week MRI scan. *Results*. There was a large statistically significant difference in time to return to work between groups. The patients with uncomplicated MTBIs had a median of 6.0 days (IQR = 0.75–14.75, range = 0–77) off work compared to a median of 36 days (IQR = 13.5–53, range = 3–315) for the complicated group. There were no significant differences between groups for any of the neurocognitive or self-report measures. There were no differences in the proportion of patients who (a) met criteria for ICD-10 postconcussional disorder or (b) had multiple low scores on the neurocognitive measures. *Conclusion*. Patients with complicated MTBIs took considerably longer to return to work. They did not perform more poorly on neurocognitive measures or report more symptoms, at 3-4 weeks after injury compared to patients with uncomplicated MTBIs.

## 1. Introduction

Most mild traumatic brain injuries (MTBIs) are not associated with visible abnormalities on structural neuroimaging. A *complicated* MTBI, in the original definition [1], was differentiated from an *uncomplicated* mild TBI by the presence of (a) a depressed skull fracture and/or (b) a trauma-related intracranial abnormality (e.g., hemorrhage, contusion, or edema). Other researchers have dropped the depressed skull fracture from the criteria and simply retained the criterion for an intracranial abnormality. The rates of complicated MTBIs, based on cohorts of patients who underwent acute computed tomography following head trauma, are presented in Table 1. The rates of abnormalities vary considerably. In general, when examining details within these studies, patients with GCS scores of 13 or 14 are more likely to have an abnormality than patients with a GCS score of 15. Other possible reasons for differences in abnormality rates could relate to technology (e.g., older scanners versus newer scanners) and referral patterns for neuroimaging (i.e., more liberal versus more conservative use of imaging).

It is seems logical to assume that worse short-, medium-, and long-term neuropsychological and functional outcome would result from complicated versus uncomplicated MTBIs. However, the results from a series of studies are mixed. As a group, patients with complicated MTBIs perform more poorly on neuropsychological tests in the first two months following injury [1–6]. These differences appear to diminish by six months following injury [7, 8]. When differences occur between groups, the effect sizes of these differences are lower than expected (i.e., medium to medium-large effect sizes or lower on a small number of tests [1–5, 7, 9]; see Borgaro and colleagues [5] for an exception). 

Some researchers have reported that patients with complicated MTBIs have worse 6–12-month functional outcome (i.e., Glasgow Outcome Scale) compared to patients who sustained uncomplicated MTBIs [1, 10, 11], and they have similar 3–5-year outcome (i.e., Functional Status Examination) as patients with a history of moderate and severe TBI [12]. There are some exceptions, however. McCauley and colleagues reported that CT abnormalities were not associated with increased risk for postconcussion syndrome at 3 months after injury [13]. Similarly, Lee and colleagues [14] reported that CT and conventional 3T MRI imaging findings do not predict neurocognitive functioning at 1 or 12 months after injury, nor functional outcome at one year after injury. It is becoming increasingly clear that complicated MTBIs represent a fairly broad spectrum of injury, with some people having very small abnormalities and excellent functional outcome and other people requiring inpatient rehabilitation and having poor outcome. 

The purpose of this study is to compare the outcome of patients with complicated versus uncomplicated MTBIs. To date, few studies have compared both neurocognitive outcome and self-reported symptoms following uncomplicated and complicated MTBI. This is a prospective study, with patients identified from the emergency department undergoing MRI and a neuropsychological evaluation at approximately 3-4 weeks after injury. It was hypothesized that patients with complicated MTBIs would report more symptoms, perform more poorly on neurocognitive testing, and take longer to return to work than patients with uncomplicated MTBIs. We hypothesized worse outcome in this group because we assume that complicated MTBIs tend to be more serious brain injuries than uncomplicated MTBIs. 

## 2. Methods

### 2.1. Participants

Participants were 47 patients with MTBIs who presented to the emergency department of Tampere University Hospital, Finland (age: M = 30.3 years, SD = 9.4, Range = 16–46; education: M = 13.0 years, SD = 2.3). The patients were selected from a larger cohort of head trauma patients enrolled in a longitudinal study, based on meeting inclusion criteria below, having complete data on all outcome measures, and having a known duration of time off work. The diagnostic criteria for MTBI used in this study were from the World Health Organization Collaborating Centre Task Force on MTBI. Inclusion criteria were as follows: (i) biomechanical force applied to the head resulting in loss or alteration of consciousness, confusion, and/or posttraumatic amnesia, (ii) loss of consciousness (LOC), if present, for less than 30 minutes, (iii) Glasgow Coma Scale (GCS) score 13–15 after 30 minutes following injury, and (iv) posttraumatic amnesia (PTA), if present, of less than 24 hours. 

Patients underwent computed tomography (CT) scanning if deemed clinically indicated, an evaluation by an ED traumatologist, and other examinations as needed. CT scanning was performed within 24 hours of admission and is used liberally for head trauma patients. Magnetic resonance imaging (MRI) was conducted at approximately three weeks after injury for research purposes, although the information was available to the patient's healthcare providers (complicated MTBI group M = 19.3, SD = 15.0, range = 1–53 days and uncomplicated MTBI group M = 25.8, SD = 5.5, range = 16–36 days). The MRI protocol included sagittal T1-weighted 3D IR prepared gradient echo, axial T2 turbo spin echo, conventional axial, and high-resolution sagittal FLAIR (fluid-attenuated inversion recovery), axial T2*, and axial SWI (susceptibility weighted imaging) series. Only trauma-related findings on CT or MRI were counted as abnormal; minor incidental findings, such as isolated white matter hyperintensities, were not considered as abnormal. Patients were excluded if significant non-trauma-related abnormalities were identified. Most were excluded due to small vessel ischemic disease, but there were also patients with multiple sclerosis, unusually large ventricles, and a history of neurosurgery.

This sample included patients (*N* = 13; 27.7%) who had an intracranial abnormality on day-of-injury CT or follow-up MRI (i.e., a complicated MTBI). None of the patients required inpatient rehabilitation. None of the patients were involved in litigation. All patients provided written informed consent according to the Declaration of Helsinki. The study protocol was approved by the Ethical Committee of the Tampere University Hospital. All patients completed self-report measures and neurocognitive testing at 3-4 weeks after injury (M = 25.8, SD = 2.9, Range 21–34 days).

### 2.2. Measures

Postconcussion symptoms were assessed using the Rivermead Post-Concussion Questionnaire (RPSQ) [15]. The RPSQ is a 16-item self-report questionnaire that measures the severity of common postoncussion symptoms on a 5-point Likert scale. The patients rated the presence of the symptoms over the past 24 hours on a scale from 0 to 4 (0 = not experienced at all after the injury, 1 = experienced but no more of a problem compared with before the injury, 2 = a mild problem, 3 = a moderate problem, and 4 = a severe problem). A total score was calculated by adding all items with a score greater than 1 (not present anymore). 

Possible depressive symptoms were assessed using the Beck Depression Inventory-Second Edition (BDI-II) [16], a 21-item self-report questionnaire. Subjects were asked to rate each item on a four-point scale ranging from zero to three. In this study, we used the total score which is the sum of all 21 items, giving a range from zero to 63. It should be noted that many symptoms on this questionnaire overlap with postconcussion symptom measured by the RPSQ. 

Self-reported fatigue was examined using the Barrow Neurological Institute Fatigue Scale (BNI-FS), an 11-item self-report questionnaire designed to assess fatigue during the early stages of recovery after brain injury [17]. Subjects were asked to rate the extent to which each of the 10 primary items has been a problem for them since the injury on a 7-point scale. Response options are as follows: 0-1 = rarely a problem; 2-3 = occasional problem, but not frequent; 4-5 = frequent problem; 6-7 = a problem most of the time. The final item (item 11) asks subjects to provide an overall rating of their level of fatigue on a scale from 0 (no problem) to 10 (severe problem). In this study the total BNI-FS score is used which is the sum of all 10 scores (min = 0, max = 70). 

General verbal intelligence was assessed with the Wechsler Adult Intelligence Scale-Third Edition (WAIS III) information subtest [18]. Learning and memory was assessed with the Rey Auditory Verbal Learning Test (RAVLT) total score (total number of words recalled in trials 1 through 5) and delayed recall (number of words recalled after 30 minutes delay) [19]. Attention and executive functioning were assessed with Stroop Color Word Test (color-word interference score, Golden version) [19], Trail Making Test (TMT) A and B (time needed to finish the task) [20], and two verbal fluency tasks: animal naming (category fluency, total number of words in one minute) and single-letter-based word generation (phonemic fluency, total number of words produced across the 3 trials) [21]. Raw scores for the neurocognitive tests were analyzed unless otherwise stated. 

## 3. Results

There were no significant differences between MTBI groups for age, education, gender, GCS score, mechanism of injury, days tested after injury, or duration of LOC, PTA, or retrograde amnesia (see Table 2). There was a large statistically significant difference in time to return to work between groups. The patients with uncomplicated MTBIs had a median of 6.0 days (mean = 12.9, SD = 18.8, IQR =.75–14.75, range = 0–77) off work compared to a median of 36 days (mean = 58.0, SD = 83.8, IQR = 13.5–53, range = 3–315) for the complicated MTBI group. 

There were no significant differences between MTBI groups for self-reported depression (BDI-II) or postconcussion symptoms (RPSQ) (all *P* > .05). There were, however, medium effect sizes for the BDI-II total score (Cohen's *d* = .52) and RPSQ total score (*d* = .43) between groups. These effects sizes suggest that the complicated MTBI group reported fewer depression symptoms and postconcussion symptoms compared to the uncomplicated MTBI group. 

There was not a statistically significant difference in the percentages of patients in the uncomplicated (44.1%) versus complicated (38.5%) MTBI groups who met ICD-10 criteria for postconcussional syndrome based on the reporting of symptoms on the RPSQ as “mild” or greater (i.e., score of 2 or higher on individual items). In contrast, 17.6% of the patients in the uncomplicated MTBI group met ICD-10 criteria for postconcussional syndrome based on the reporting of symptoms on the RPSQ as “moderate” or greater (i.e., score of 3 or higher on individual items); no patients in the complicated MTBI group met this criterion for the syndrome. 

As seen in Table 3, there were no significant differences between MTBI groups on the neuropsychological tests (all *P* > .05). Although not significantly different, medium effect sizes were found for the RAVLT total score (*d* = .39), RAVLT delayed recall score (*d* = .47), Animal Naming test (*d* = .43), and Stroop Color-Word (*d* = .47). Paradoxically, the complicated MTBI group performed better on verbal learning and memory (RAVLT) and executive functioning (Stroop), but worse on a test of verbal fluency (Animal Naming). 

The prevalence of low scores across the battery of cognitive tests was determined for each group (i.e., eight scores (not including the Rey Copy) derived from the six tests were considered simultaneously). A low score on the neurocognitive tests was defined as falling below the 10th percentile compared to published normative data. There was not a significant difference in the percentage of patients with uncomplicated (26.5%) versus complicated (23.1%) MTBIs who had two or more low scores, when eight test scores were considered simultaneously. 

## 4. Discussion

A substantial minority of patients who sustain an MTBI and are evaluated in an emergency department will have a visible abnormality on early CT scanning (Table 1). These patients are conceptualized as having a complicated MTBI. Researchers have reported that those with complicated MTBIs, as a group, are more likely to have early cognitive deficits [1–6] and worse medium [1, 10, 11] and long-term [12] functional outcome. In contrast, however, some researchers have not found important differences between those with complicated versus uncomplicated MTBIs. For example, in one study, patients with complicated MTBIs were not more likely to have a postconcussion syndrome at three months after injury [13], and another research group reported that neuroimaging findings did not predict cognitive functioning at one or 12 months after injury, or functional outcome at one year after injury [14].

In the present study, as hypothesized, patients with complicated MTBIs took longer to return to work. It has been noted that most patients return to work after injury despite having some symptoms [22]. In this study, we did not explicitly examine if the patients had symptoms prior to returning to work. Yet, the majority of the study population had returned to work by the time of the neuropsychological assessment. Therefore, their self-reported symptoms at the time of evaluation likely reflect their situation after returning to work, supporting the idea that it is common to return to work while still having some symptoms. One possible reason for why intracranial lesions were correlated with longer time off work may be that doctors are likely to grant longer sick leaves when there is objective evidence of brain injury; in that case the duration of the postinjury sick leave might reflect, in part, the behavior of doctors in the Finnish system. We have no way of determining whether, or not, doctors in the community are likely to grant longer sick leaves to patients with complicated MTBIs, but we have anecdotal evidence from emergency department physicians that they tend to prescribe longer initial periods of leave for patients with more serious injuries such as those with intracranial abnormalities. 

In the present study, the patients with complicated versus uncomplicated MTBIs were compared on neurocognitive testing and symptoms ratings at approximately 3-4 weeks after injury. It was hypothesized that those with complicated MTBIs would perform more poorly on cognitive testing and report more symptoms than those who did not have imaging abnormalities. Contrary to these hypotheses, there were no differences between the two groups on neurocognitive testing or symptom reporting. Surprisingly, there were trends toward those with complicated MTBIs reporting fewer symptoms and performing somewhat better on cognitive testing. 

There are methodological differences and limitations with the present study, in comparison to previous studies, that might have influenced the results. First, there were a small number of subjects in this study that were identified as having imaging abnormalities; thus, the statistical analyses were underpowered. However, when examining the means and SDs, there was not a trend toward greater symptoms or worse cognitive test performance in the complicated MTBI group. In fact, there were trends toward fewer symptoms and better performance in this group. This reduces the likelihood that the present findings represent a Type 2 statistical error. Second, most previous studies classified patients as having complicated MTBIs based on day-of-injury CT scanning only, and some of these studies are older and the CT technology might have been less refined. The present study identified subjects based on CT and MRI, with some of our sample not showing day-of-injury CT abnormalities—only abnormalities on MRI. Third, some previous studies have included subjects with complicated MTBIs who required inpatient rehabilitation. None of the present patients were injured that badly. Finally, some previous studies have included patients in litigation, whereas no patients in this study were involved in litigation. Therefore, it is possible that the present group of patients with complicated MTBIs were less severely injured than some of the samples from previous studies.

**Figure 1:**
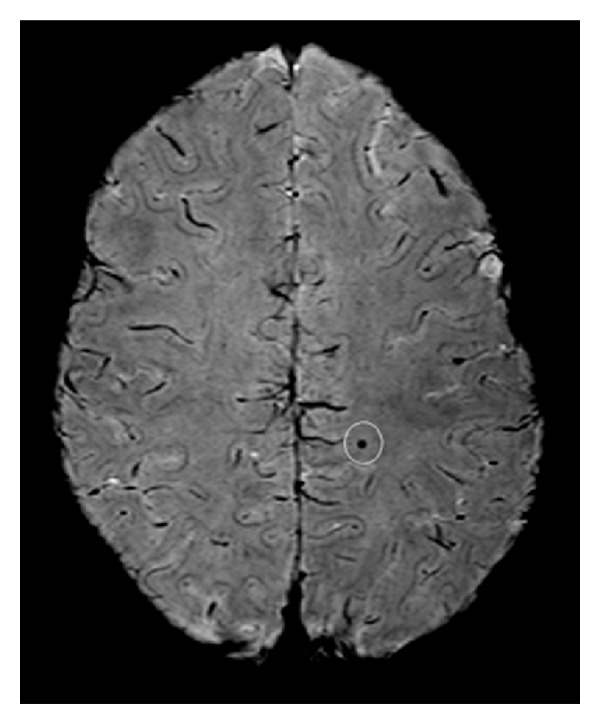
Hemosiderin detected with multiecho susceptibility weighted imaging using a 3 Tesla scanner. Multiecho SWI image (Philips Achieva 3T; 5 echoes; voxel size = 0.32 × 0.32 × 0.75 mm^3^). Courtesy of Alexander Rauscher, Ph.D., UBC MRI Research Centre, Department of Radiology, University of British Columbia, Vancouver, Canada.

As technology evolves, some researchers will be tempted to broaden the criteria for complicated MTBI. In the past, intracranial abnormalities were identified using CT or conventional MRI. With advancements in technology, smaller and smaller abnormalities can be detected using structural imaging. For example, the area of hemosiderin (iron-rich staining of tissue from an area with past blood) shown in Figure 1 using multiecho susceptibility weighted imaging (SWI) with 5 echoes [23, 24] on a 3 Tesla MRI scanner would be undetectable with a modern CT scan and would likely be missed using 1.5 or 3.0 Tesla MRI conventional sequences [25]. Therefore, in past studies this subject would be classified as having an uncomplicated MTBI, but in future studies this abnormality might qualify for classification as a complicated MTBI. However, this subject was actually a healthy control subject in one of our studies. He had no known history of an injury to his brain. Thus, not only might the criteria for a complicated MTBI evolve to include smaller and smaller abnormalities—but some of these abnormalities might not be related to the MTBI—thus resulting in misdiagnosis. 

In conclusion, patients with complicated MTBIs took longer to return to work. They did not, however, perform more poorly on neurocognitive measures or report more symptoms, at 3-4 weeks after injury compared to those with uncomplicated MTBIs. As the literature evolves, it is becoming clear that complicated MTBIs represent a broad spectrum of injury, with some people having very small abnormalities and excellent functional outcome, other people requiring inpatient rehabilitation and having poor outcome, and a diverse set of outcomes in between. 

## Figures and Tables

**Table 1:** Rates of complicated mild TBI in adults.

First author	Year	Country	Total *N*	Number Scanned	GCS scores	% Abnormal
Livingston [26]	1991	USA	111	111	14-15	14
Stein [27]	1992	USA	1,538	1,538	13–15	17.2
Jeret [28]	1993	USA	712	702	15	9.4
Moran [29]	1994	USA	200	96	13–15	8.3
Borczuk [30]	1995	USA	1,448	1,448	13–15	8.2
Iverson [31]	2000	USA	912	912	13–15	15.8
Thiruppathy [32]	2004	India	381	381	13–15	38.9
Stiell [33]	2005	Canada	2,707	2,171	13–15	12.1
Stiell [33]	2005	Canada	1,822	1,822	15	8.0
Ono [34]	2007	Japan	1,064	1,064	14-15	4.7
Saboori [35]	2007	Iran	682	682	15	6.7

**Table 2:** Demographic and injury severity characteristics.

	Uncomplicated MTBI		Complicated MTBI			
	M	SD	M	SD	*p*	*d*
Age (in years)	30.8	9.0	29.2	10.9	0.601	.17
Education (in years)	13.1	2.3	12.9	2.3	0.859	.06
WAIS-III information (SS)	10.9	2.4	10.4	1.4	0.492	.23
GCS score in ED	14.9	0.3	14.9	0.3	0.693	.13
Days tested after Injury	26.0	2.9	25.4	3.0	0.523	.21
Duration of loss of consciousness (minutes)	0.6	1.5	1.0	1.5	0.514	.25
Duration of posttraumatic amnesia (minutes)	333.0	448.2	366.7	420.8	0.822	.08
Duration of retrograde amnesia (minutes)	9.2	23.6	21.5	66.6	0.354	.34
Number of days to return to work	12.9	18.8	58.0	83.8	0.002	1.2

	*f*	%	*f*	%	*χ* ^2^	

Gender						
Male	17	50.0	7	53.8	0.813	—
Mechanism of injury						
MVA	15	44.1	5	38.5	0.726	—
Other bodily injuries	9	26.5	4	30.8	0.768	—
CT: day of injury						
Abnormal	0	0	8	61.5	—	—
MRI: 3 weeks after						
Abnormal	0	0	12	92.3	—	—
Not available	0	0	1	7.7		

Note: *N* = 47 (uncomplicated MTBI, *n* = 34; complicated MTBI, *n* = 13). Cohen's effect size (d): small (.20), medium (.50), large (.80). CT= computed tomography; MRI:magnetic resonance imaging; GCS: Glasgow Coma Scale; MTBI: mild traumatic brain injury; MVA: motor vehicle accident. A Mann-Whitney *U* test was used for the number of days to return to work comparison.

**Table 3:** Descriptive statistics (raw scores) and effect sizes: self-report and neurocognitive tests.

	Uncomplicated MTBI		Complicated MTBI			
	M	SD	M	SD	*p*	*d*
Self-report Measures						
BDI-II total	7.6	6.7	4.5	4.0	.130	.52
RPSQ total	11.6	11.8	7.2	6.2	.358	.43
Barrow Fatigue Scale	16.4	15.3	13.0	9.7	.464	.25
Neurocognitive Tests						
RAVLT total	56.9	7.8	60.2	10.1	.237	.39
RAVLT delay	11.1	2.8	12.4	2.6	.159	.47
RCFT copy	35.7	0.7	35.6	0.8	.792	.09
RCFT Immediate	25.4	5.9	23.8	5.2	.402	.28
Phonemic Fluency total	38.7	9.4	39.8	13.4	.766	.10
Animal Naming total	25.1	6.4	22.5	5.0	.194	.43
Trails A (in seconds)	27.3	9.5	28.5	8.7	.685	.13
Trails B (in seconds)	62.5	24.8	57.5	13.3	.495	.23
Stroop Color-Word	42.1	8.2	45.9	7.2	.157	.47

Note: *N* = 47 (uncomplicated MTBI, *n* = 34; complicated MTBI, *n* = 13); Cohen's effect size (*d*): small (.20), medium (.50), large (.80). BDI-II: Beck Depression Inventory-Second Edition; RPSQ: Rivermead Postconcussion Scale; RCFT: Rey Complex Figure Test; RAVLT:Rey Auditory Verbal Learning Test; MTBI:mild traumatic brain injury.
